# The “Health Coaching” programme: a new patient-centred and visually supported approach for health behaviour change in primary care

**DOI:** 10.1186/1471-2296-14-100

**Published:** 2013-07-17

**Authors:** Stefan Neuner-Jehle, Margareta Schmid, Ueli Grüninger

**Affiliations:** 1 Institute of General Practice and Health Services Research, University of Zürich, Pestalozzistrasse 24, Zürich 8091, Switzerland; 2 Institute of Social and Preventive Medicine, University of Zürich, Zürich 8091, Switzerland; 3 Swiss College of Primary Care Medicine, Bern, Switzerland

**Keywords:** Health behaviour, Short intervention, Motivational interviewing, Family medicine, Primary care, Counselling, Patient-centredness, Health promotion

## Abstract

**Background:**

Health related behaviour is an important determinant of chronic disease, with a high impact on public health. Motivating and assisting people to change their unfavourable health behaviour is thus a major challenge for health professionals. The objective of the study was to develop a structured programme of counselling in primary care practice, and to test its feasibility and acceptance among general practitioners (GPs) and their patients.

**Methods:**

Our new concept integrates change of roles, shared responsibility, patient-centredness, and modern communication techniques—such as motivational interviewing. A new colour-coded visual communication tool is used for the purpose of leading through the 4-step counselling process. As doctors’ communication skills are crucial, communication training is a mandatory part of the programme. We tested the feasibility and acceptance of the “Health Coaching” programme with 20 GPs and 1045 patients, using questionnaires and semistructured interviewing techniques. The main outcomes were participation rates; the duration of counselling; patients’ self-rated behavioural change in their areas of choice; and ratings of motivational, conceptual, acceptance, and feasibility issues.

**Results:**

In total, 37% (n=350) of the patients enrolled in step 1 completed the entire 4-Step counselling process, with each step taking 8–22 minutes. 50% of ratings (n=303) improved by one or two categories in the three-colour circle, and the proportion of favourable health behaviour ratings increased from 9% to 39%. The ratings for motivation, concept, acceptance, and feasibility of the “Health Coaching” programme were consistently high.

**Conclusions:**

Our innovative, patient-centred counselling programme for health behaviour change was well accepted and feasible among patients and physicians in a primary care setting. Randomised controlled studies will have to establish cost-effectiveness and promote dissemination.

## Background

In addition to non-modifiable factors—such as genetic disposition, sex, age, and ethnicity—*health related behaviour* is an important determinant of chronic disease (for example, coronary heart disease, cardiovascular disease (CVD), and stroke), with a high impact on the evolution and course of the disease [1]. CVD is a major issue in public health, contributing excessively to the overall morbidity and mortality in the populations of industrialised societies [2]. This highlights an urgent need for action to diminish the burden of disease. Other important diseases—such as malignancy, dementia, and diabetes—are also associated at least partially, owing to behaviourally modifiable risk factors. The most relevant areas of health related behaviour are therefore dietary habits and body weight control, physical activity, smoking, alcohol consumption, and psychosocial stress [1].

Motivating and assisting people to change their unfavourable health behaviour is a major challenge for health professionals. Growing evidence suggests that *involving people in decision-making* is fostering their sense of self-determination, self-responsibility, and ownership, and has positive effects in terms of their motivation, satisfaction, adherence to an intervention, and even health outcomes [3,4]. Patients increasingly seek more active participation in healthcare decisions, albeit not all of them to the same degree [5]. Experts have called for a shift towards a meaningful dialogue between patients and physicians and shared decision making [6].

In the well-known transtheoretical model of behaviour change proposed by Prochaska and DiClemente [7], a change of behaviour requires awareness and knowledge of the relevant problem as necessary prerequisites. Informing persons at risk in the optimal way, in order to create motivation, is not easy and requires attention. In addition to using words and numbers to explain risk, visual communication tools seem to improve the understanding of risk and to increase self-efficacy while dealing with risk [8,9].

### Concept

#### Concept of the “Health Coaching” project

Since 2006, a task force of the Swiss College of Primary Care Medicine developed a programme for behavioural counselling and health promotion in primary care. The innovative components of this programme are as follows:

-*A change of roles and sharing responsibility between doctor and patient*: Patient and general practitioner (GP) are a team. As a coach, the GP transfers the responsibility for health partly to the patient, who becomes the main actor for her/his health. Patients will be planning and implementing their own health projects step by step, based on their own preferences and experiences. If necessary, other professionals and services can be included, for example, practice staff or third party counsellors.

-*Patient-centred choice of the area of action*: Programmes have traditionally focused on *one* topic—for example, weight control, alcohol consumption, or smoking cessation. The “Health Coaching“ programme offers a choice of six topics that are crucial for health, either at an individual level or from a public health perspective (these are the six most important behavioural contributors to the burden of disease in Switzerland): dietary habits and body weight control, physical activity, smoking, alcohol consumption, and psychosocial stress [1]. Out of these six, the patient – who is at the centre – can choose according to her/his preferences and subjectively perceived needs. Additionally, patients may choose other topics—for example, sleep deprivation—as other, potentially harmful, health-related behaviours.

-The counselling techniques are based on *modern communication concepts specifically operationalised for use in office consultations: health literacy* and *patient empowerment*[10], *shared decision making*[11], *transtheoretical model of behaviour change (TTM)*[7], counselling based on motivational stages [12], *motivational interviewing*[13], and various risk communication formats and models [14].

-*GP training courses* (communication skills, especially motivational interviewing techniques) are mandatory, as the change of role and communication techniques are beyond the traditional patterns of GPs’ training and professional work. Analogous to the stepwise counselling with patients (see below), the training courses are organised stepwise: a. sensitisation workshops (2–3 hrs), b. skills training courses (2×1 day, with standardised patients), and c. feedback sessions to share experiences.

#### The concept of the pictorial tool

**Figure 1:**
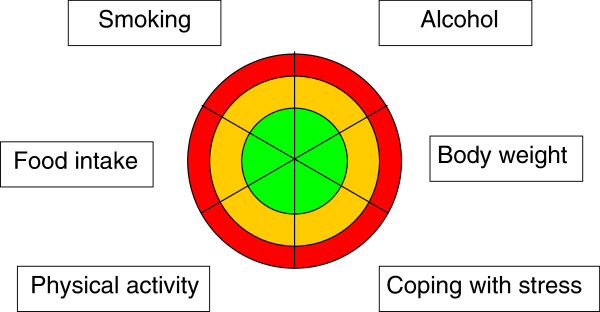
**The visual tool to initiate discussion, help with decisions, and visualise progress: a colour-coded circle with six areas relevant for health.** Colour code: red = unfavourable health behaviour, orange = health behaviour could be improved, green = favourable health behaviour. Participants define their positions within the areas, set a mark, and choose their goal.

The risk visualisation tool (Figure 1) is the central communication element of the programme. It includes categorisation by colour coding to suggest safety or danger to patients at baseline. It facilitates a choice of the six potential areas of action, as a comparison with other areas is possible at one glance. During counselling, repeated use of the tool visualises for the patient the *change of behaviour over time*: “Am I successful in changing my behaviour (moving from an unfavourable outer zone in the circle to a more favourable, more central zone in the circle), or not yet?” The current category of risk at baseline and its development during the “personal project” are discussed by GPs and patients by using a combination of verbal and visual communication formats. According to the recent literature, such a combination of different communication formats is necessary for optimal understanding [15,16].

### Counselling step by step

**Figure 2:**
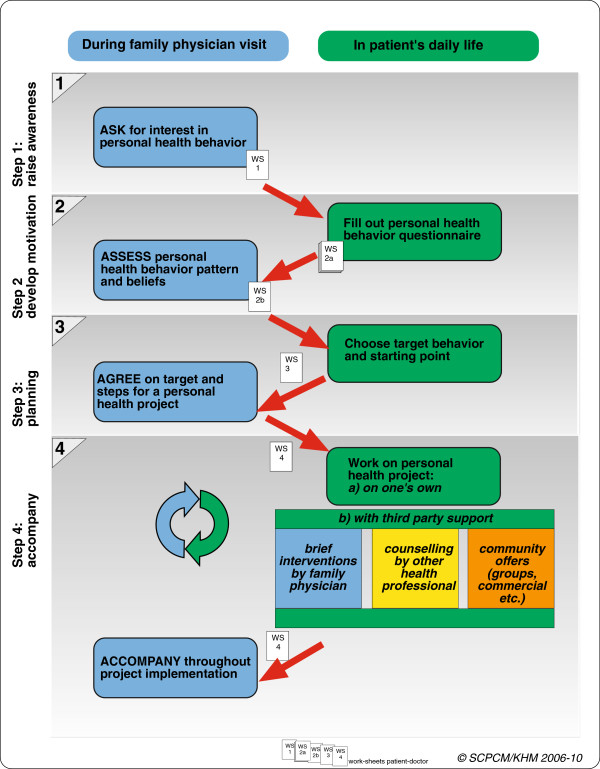
Stepwise counselling of the “Health Coaching“ programme.

Each step of counselling in the Health Coaching programme refers to a step of change in awareness, motivation, or action on the part of patient, in the sense of the TTM [7] stages of behavioural change (Figure 2). This process is based on the fact that on beginning counselling, people are at various stages of change or readiness for a change:

•Step 1 - *sensitize*: GPs introduce a change of roles and responsibilities. For example, GPs may switch from the paternal role to the coaching role, asking: *“Until now, we have been talking about what I can do to help with your illness. Now, I’m interested to know what you want to do for your own health—is that okay with you?”* If patients agree, they will assess their own health behaviour and motivation to change.

In this initial session the decisive change of focus and distribution of role and responsibility are best introduced by the patient’s physician. Later, the counselling may be supported and/or continued by other qualified medical personnel such as trained practice staff.

•Step 2 – *create and promote motivation*: Health behaviour is analysed by using a questionnaire, to quantify behavioural risks in the areas of choice, and combined with a comment of the GP about the findings.

•Step 3 – *plan:* On the basis of these objective findings, patients choose their topic, define a specific goal, and develop steps for how to get there (thereby designing their personal health project). GPs frame the need for change in a positive way: as an opportunity for improvement rather than for avoiding risks.

•Step 4 – *action and coaching*: Patients implement the plan, dealing with barriers and resources. In follow-up consultations, patients and GPs evaluate progress in the patient’s health programme, adapt the programme, and make changes as needed.

## Methods

### Feasibility and acceptance study

**Figure 3:**
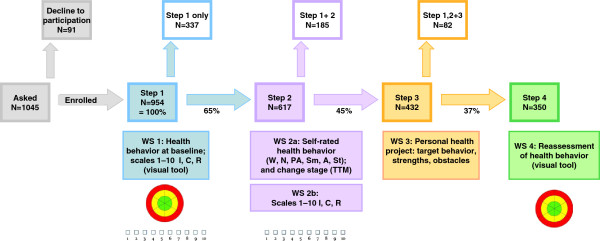
**Process steps, patient participation, and continuation rate from one step to the next step, and the times of data collection.** Abbreviations: WS: work sheet (one worksheet per step): WS 1: Assessing awareness (self-rated health behaviour at baseline, using visual tool and visual analogue scales); WS 2: Exploring motivation (with extended questionnaire about health behaviour in the six areas and health-related attitudes); WS 3: choice of target behaviour and action plan; WS 4: evaluation of success (visual tool). I: importance. C: Confidence. R: Readiness. W: Weight. N: Nutrition PA: Physical Activity St:Stress Sm: Smoking A:Alcohol. TTM: Transtheoretical Model.

In 2009–10, we conducted a feasibility and acceptance study with an extended evaluation to prove these concepts in a canton (Swiss state/district) in eastern Switzerland. After approval by the ethical committee of the Canton St Gallen, we enrolled 20 GPs (by postal invitation), who in turn recruited 1045 patients into the study during a 12-month period. Written, informed consent for participation in the study was obtained from participants. The mean age of patients was 50 (range 15–75) years, 53% were men. Participants’ health behaviours in the six areas and educational attainments were close to those of the average population in Switzerland. Figure 3 shows the workflow of counselling, rates of patient participation from one step to the next, and the times of data collection. We collected data from the patient-doctor worksheets used in the office consultations, patient questionnaires after counselling, office log books documenting the duration of counselling sessions, participants’ questionnaires at different times in the process, GPs’ questionnaires, semistructured interviews with GPs at the end of the 12 month study period, and GPs’ group interviews during the training courses and feedback meetings. We used SPSS (Statistical Software Package, Version 19, SPSS Inc., Chicago, Illinois) for our statistical analysis, and we calculated means, medians, standard deviations (SDs), and interquartile ranges (IQRs). The qualitative data were categorised based on thematic content analysis.

## Results

Participation rates were 91% for Step 1 and 65%, 70%, and 81% for Steps two, three, and four, respectively. In total, 37% (n=350) of the patients enrolled in Step 1 completed the 4-step counselling process. On average, every GP undertook counselling with 45 patients within this 12 month period, with a mean duration of 7.7 minutes for Step 1 (SD 4.2, median 6, IQR 5–10), 21.9 minutes for Step 2 (SD 9.5, median 20, IQR 15–30), 20.3 minutes for Step 3 (SD 8.8, median 20, IQR 15–25), and 18.2 minutes for Step 4 (SD 8.8, median 16, IQR 12–20).

The self-rated importance, self-confidence (to reach the goal), and readiness to change were 8.3 (SD 1.8), 7.3 (SD 2.1), and 7.8 (SD 2.1) points on a 10-point Likert scale at the beginning, and increased throughout the counselling. One third of 528 targets chosen by participants at Step 3 related to weight control and 20% to physical activity. Eating habits, tobacco smoking and coping with stress accounted for 12 to 13% each, alcohol consumption for 2%, and other goals (symptom control, wellbeing, or better health generally) for 8%. Acceptance and feasibility rating by patients were generally high (between 3.3 and 3.8 points on a 4-point Likert scale, SD 0.44-0.77) for concept, materials, the role of the GP, and overall usefulness (Table 1). Quantitative and qualitative data from GPs show similarly positive feedback: the new, more relaxed approach to the patient improved work quality and satisfaction. GPs felt relieved to hand over responsibility and share it with the patients. The “Health Coaching” programme seems to empower GPs to address health related issues during patient encounters. After the end of the 12 month study period, 16 of the 20 study GPs spontaneously expressed that they wanted to continue with the programme.

**Table 1:** Items and ratings of participants in regard to acceptance and feasibility; rating on a 4-point-Likert scale: Category 1=strongly disagree, 2=somewhat disagree, 3=somewhat agree, 4=strongly agree

**Item in the questionnaire**	**mean**	**SD**	**Cat. 1**	**Cat. 2**	**Cat. 3**	**Cat. 4**
I appreciate that my GP asked me to join the program.	3.7	0.56	1%	2%	20%	76.4%
I appreciate my GP’s support in improving my health.	3.8	0.44	0.3%	1.1%	16.9%	81.7%
It is important that I myself can do something to improve my health.	3.8	0.48	0%	1.8%	21.8%	76.4%
The procedure using work sheets is useful and meets my current needs.	3.4	0.62	0.4%	5.7%	43.3%	50.6%
The three-coloured circle was useful in defining for myself my own position regarding the different health behaviours.	3.5	0.65	1.4%	4.8%	39.4%	54.4%
The questionnaire helped me see where I could improve my health behaviour.	3.5	0.64	1%	5%	36.6%	57.4%
My doctor’s regular feedback improved my motivation to stick to my goals.	3.4	0.71	1.9%	7.6%	39.7%	50.8%
My doctor took enough time to talk to me about the work sheets.	3.7	0.64	1.8%	3.7%	21.7%	72.8%
My doctor was always concerned about my needs during the discussions.	3.7	0.47	0%	1.1%	25.3%	73.6%
I was able to discuss my most important health issues with my doctor.	3.8	0.48	0%	2.3%	18.9%	78.8%
Since then, I have become more confident that I am able to change my own health behaviour (to have an influence on my health, respectively).	3.3	0.69	1%	9.5%	47.6%	41.9%
My doctor’s support has increased my self confidence that I am able to achieve a change.	3.5	0.63	0%	7.4%	36.8%	55.8%
For me, participating in the “Health Coaching“ programme has been worth while.	3.4	0.77	2.3%	10.6%	29.9%	57.1%
I think the “Health Coaching“ programme should be part of a GP’s standard practice offerings.	3.6	0.66	1.1%	5.9%	30.6%	62.5%

The pictorial tool was rated 3.5 (SD 0.65) in terms of being helpful and practicable, and higher in Steps 3 and 4 of counselling (3.6, SD 0.55). Similarly, GPs gave positive feedback regarding the tool, considering it to be a helpful schematic and vehicle for counselling in terms of conveying information, stimulating awareness, and visualising comparisons across behaviours and changes of these.

**Figure 4:**
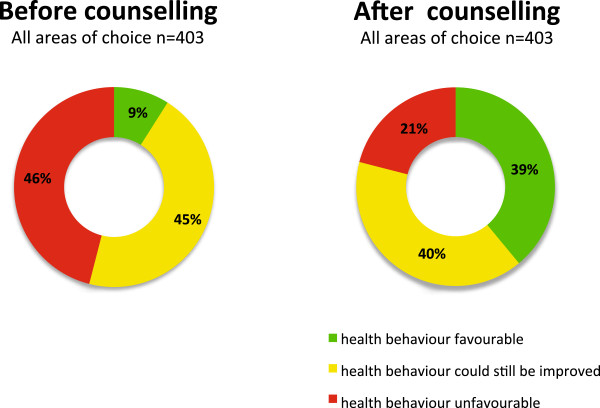
**Proportion of participants in the three possible categories of health behaviour, in their area of choice, before and after counselling (self-ratings).** Numbers before and after counselling are not consistent because of discontinuation of the stepwise counselling procedure and missed items. Numbers refer to targets (areas of choice), not to participants.

Some 46% of participants’ health behaviour ratings (n=403) in their targeted area of choice at Step 1 defined in the unfavourable category (red area), compared with 21% after counselling (n=303), and 9% defined in the favourable category (green area), compared with 39% after counselling (Figure 4). Among participants who completed the counselling programme, 50% of ratings (n=303) improved by one or two categories in the three-colour circle, 43% of ratings did not change category, and 7% deteriorated by one category. The proportions differed to a small degree, depending on the targeted area chosen (Table 2). Among participants without subjective behavioural improvement, most experienced a subjective benefit from counselling, regardless, as measured by indices of attributed importance, self-confidence, and stage of readiness: *–“I have learned that this is not about my blood pressure, but about me as a person, and that it is me who is responsible for my health, and that I can’t delegate this to my physician”. – “Being more active can be fun.“ – “I often remember what I discussed with my GP”.* Participants increased their knowledge and awareness of their health behaviours and learned to seek support, if necessary.

**Table 2:** Change in self-defined categories of health behaviour, in the six areas of choice, after completing the four-step counselling procedure

Target (area of choice)*	**Improvement by 1-2 categories**	**No change of categories**	**Deterioration by 1-2 categories**
Body weight n=106	43%	53%	4%
Physical activity n=75	52%	35%	13%
Eating pattern n=42	49%	46%	5%
Coping with stress n=38	58%	34%	8%
Smoking n=35	60%	37%	3%
Alcohol consumption n=7	57%	43%	0%
**Total n=303**	**50%**	**43%**	**7%**

*Numbers of targets (areas of choice) relate to data from 231 participants who completed Step 4, which is equivalent to 303 targets (several participants defined more than one target for their personal project). Of all participants completing Step 4 (n=350), data on these Step 4 target ratings were missing in 119 participants, as the visual tool in Step 4 was designed as counselling support and not primarily as data collection tool.

## Discussion

To our knowledge, the “Health Coaching” project is the first multidimensional, patient-centred and systematic approach to foster GPs’ counselling about health relevant behaviour. The high participation rate at baseline and the above-average adherence rates throughout the 4-step counselling process are strong indicators of acceptance and feasibility of our approach, as are the high ratings related to these topics from the patients’ and GPs’ questionnaires and interviews. For GPs, 1–2 starting sessions and 2–3 complementary (Step 2–4) sessions per week should present an acceptable supplementary workload in their practices, and the counselling times (8 to 22 minutes) still meet the criteria of a short intervention and cost-effectiveness.

We were encouraged by the patients’ high self-ratings of motivation and readiness to change at baseline. The Health Coaching programme appears to have met patients’ needs, traditionally unmet and undervalued, with respect to discussing health promotion topics with their GPs. The fact that the ratings of importance, self-confidence (to reach the goal), and readiness to change increased during counselling underlines the efficacy of our approach, although selection bias might limit the validity of these findings (see below).

The use of colours in our pictorial tool provided patients with an important reference point regarding the severity of risk. In this colour coding, the colour red denotes urgency (even danger) and the necessity to discontinue the behaviour, whereas the green colour denotes “no risk” [17]. This coding system relies on what most of us learn early in our childhood: the symbolism of traffic light colours. In the meantime, many risk calculators are using colour-coded tables or output categories [18]; consequently, this method of communication has become familiar. Patients seem to feel a need to compare themselves to an average value (or population) [19], and the result may improve their motivation to change, or, equally important, when they report being in the healthy (green) range, this can help them to sustain existing or recently acquired beneficial habits.

The main finding is an improvement in self-rated health behaviour by at least one of two possible levels in half of the participants in the Health Coaching programme; those showing no behavioural improvement reported some benefit as well. This benefit translates into an increase in awareness, perceived self-confidence, and readiness to take responsibility. In comparison to other preventive interventions, the “number needed to treat” (NNT) in our intervention to achieve a successful change of health behaviour, is low. In order to change behaviour successfully in one patient, we had to invite six patients to participate (one in three participants completed the four counselling steps, and one in two completers changed their behaviour successfully). The positive effects observed in the participants who did not complete the programme or in participants who did not report improvement are additional positive outcomes; this further improves the effort to benefit ratio.

Although acceptance and feasibility were high, research is needed to elucidate barriers among patients and GPs against the use of programmes similar to our’s, and to identify factors that may promote and facilitate this sort of approach. We suggest exploratory studies with focus groups, and interviewing techniques focusing on these factors. More studies with a randomised controlled design and a longer follow-up period are needed to establish objective and clinically relevant outcomes. Finally, the cost effectiveness of the Health Coaching programme will have to be investigated, by means of health services research, for example, on how biomedical or surgical interventions can be avoided by successful health behaviour changes in response to counselling.

In sum, our programme is innovative and atient-centred. It appeared to be well accepted by patients and GPs, and highly feasible in a primary care setting. To publicise this approach and programme among GPs, several issues need to be addressed. These include the smooth integration into busy office schedules and doctors’ workload, as well as the introduction of appropriate reimbursement for the counselling sessions. Sharing counselling activities with other health professionals, e.g. practice staff, may be one way to facilitate this. The extension of health behaviour change competencies is necessary at various levels: in the education of physicians (undergraduate and postgraduate training, as well as in continuous medical education); in practice-based research; in medical associations, in order to recognise these skills as basic medical competencies; and in the support of health policymakers at the legislative, executive, and regulatory levels. Finally, extending our Health Coaching programme to other healthcare professionals, including non academic professionals, is a promising option in order to promote its effects. Feasibility studies to explore this topic and access to it are necessary [20].

### Limitations

Our feasibility and acceptability study was run in one region of Switzerland, with a relatively small number of GPs; therefore, the results have limited generalisability. Most outcomes were self-reports rather than clinical outcomes, as we did not have the intention nor the means to conduct a randomised controlled trial to measure the clinical effects of the intervention, but, rather, to test our approach and its acceptance and feasibility.

We cannot exclude selection bias owing to the way in which GPs and patients were recruited: GPs with a higher motivation for counselling activities in health behaviour may have been more inclined to accept the invitation, and patients willing to participate may have been more motivated to start counselling and undertake activities to change their behaviour than those who declined. The unexpectedly high rates of motivation (preparation stage of the TTM model) for a change at baseline may be an indicator of a possible bias. However, the fact that only 9% of invited patients declined participation minimises this possible bias, and the proportion of invited patients who highly appreciated a discussion about their health behaviour with their GPs (three out of four) was not significantly different from an average European general practice population [21]. Without a randomised controlled study, it is difficult to estimate the size of this selection bias, and the main focus of our study was the feasibility and acceptance.

In regard to the pictorial risk communication tool, it was not our intention to validate the tool *independently* of the counselling effect. A full validation would require a randomised controlled design and a separation of communication tool and counselling as interventions. Regardless, we included patient ratings and GPs’ comments about the pictorial tool in the evaluation.

## Conclusions

To our knowledge, our “Health Coaching” programme is the first multidimensional, patient-centred and systematic programme that has been designed to promote GPs’ counselling about health-related behaviour, and has been shown to be feasible and acceptable to patients in a primary care setting.

Further studies are needed including randomised controlled trials to examine the cost effectiveness of health coaching by GPs, with measurable and clinically relevant outcomes. The results will hopefully encourage stakeholders and politicians to take responsibility for supporting health promoting programmes in primary care. This may facilitate dissemination, in order to improve health related behaviours at an individual and public health level, thereby helping to reduce the burden of non-communicable diseases. In the face of these diseases, such programmes may be singularly important in reversing this epidemic.

## Abbreviations

CVD: Cardiovascular disease; GP: General practitioner; IQR: Interquartile range; NNT: Number needed to treat; RCT: Randomised controlled trial; SD: Standard deviation; TTM: Transtheoretical model of behaviour change.

## Competing interests

The authors declared that they have no conflict of interest.

## Authors’ contributions

UG, SNJ, and MS developed the concept and designed the feasibility and acceptance study. MS conceived the evaluation process, carried out the data collection, and performed the statistical analysis. SNJ drafted the manuscript. All authors contributed to the writing of the manuscript, and all authors read and approved the final manuscript.

## Pre-publication history

The pre-publication history for this paper can be accessed here:

http://www.biomedcentral.com/1471-2296/14/100/prepub
